# Up-regulation of neogenin-1 increases cell proliferation and motility in gastric cancer

**DOI:** 10.18632/oncotarget.1960

**Published:** 2014-05-13

**Authors:** Seok-Jun Kim, Yuan-Guo Wang, Hyun-Woo Lee, Hyeok Gu Kang, Sun-Hyuk La, Il Ju Choi, Tatsuro Irimura, Jae Y. Ro, Robert S. Bresalier, Kyung-Hee Chun

**Affiliations:** ^1^ Department of Biochemistry and Molecular Biology, Yonsei University College of Medicine, Seoul, Republic of Korea; ^2^ Minimally Invasive Tumor Therapy Laboratory, Radiology Department, Beth Israel Deaconess Medical Center, Boston, MA, USA; ^3^ Department of Biochemistry, College of Life Science and Biotechnology, Yonsei University, Seoul, Republic of Korea; ^4^ Center for Gastric Cancer, Division of Translational & Clinical Research I, National Cancer Center, Gyeonggi-do, Republic of Korea; ^5^ Institute of medical innovation, St. Luke's international Hospital, Chuo-ku, Tokyo, Japan; ^6^ The Methodist Hospital, Department of Pathology and Genomic Medicine, Weil Medical College of Cornell University, Houston, TX, USA; ^7^ Department of Gastroenterology, Hepatology, and Nutrition, The University of Texas M.D. Anderson Cancer Center, Houston, TX, USA; ^8^ Brain Korea 21 PLUS Project for Medical Science, Yonsei University

**Keywords:** Neogenin-1, Galectin-3, Heat shock factor (HSF)-1, Cancer metastasis, Gastric cancer

## Abstract

Although elevated expression of neogenin-1 has been detected in human gastric cancer tissue, its role in gastric tumorigenesis remains unclear due to the lack of neogenin-1 studies in cancer. Therefore, we demonstrated here the function and regulatory mechanism of neogenin-1 in gastric cancer. Neogenin-1 ablation decreased proliferation and migration of gastric cancer cells, whereas its over-expression reversed these effects. Xenografted analyses using gastric cancer cells displayed statistically significant inhibition of tumor growth by neogenin-1 depletion. Interestingly, galectin-3 interacted with HSF-1 directly, which facilitated nuclear-localization and binding on neogenin-1 promoter to drive its transcription and gastric cancer cell motility. The galectin-3-increased gastric cancer cell motility was down-regulated by HSF-1 depletion. Moreover, the parallel expression patterns of galectin-3 and neogenin-1, as well as those of HSF-1 and neogenin-1, were detected in the malignant tissues of gastric cancer patients. Taken together, high-expression of neogenin-1 promotes gastric cancer proliferation and motility and its expression is regulated by HSF-1 and galectin-3 interaction. In addition, we propose further studies for neogenin-1 and its associated pathways to provide them as a proper target for gastric cancer therapy.

## INTRODUCTION

Gastric cancer is the second most common cause of cancer-related death in the world [1]. Despite recent advances in treatments involving surgery, chemotherapy, and radiation therapy, gastric cancer is difficult to cure unless it is found at an early stage [2]. Even patients who present with the most favorable characteristics and who undergo curative surgical resection often die of recurrent disease due to metastasis and drug resistance [3]. Therefore, to cure gastric cancer, we need to understand the molecular mechanisms behind gastric cancer metastasis and drug resistance.

Through previous studies (GSE 29630), we reported microarray data on galectin-3 silencing in gastric cancer cell lines [4] which indicated that several cell motility-related genes changed [5-8]. Among these changes, in the present study we focus on neogenin-1 expression. Neogenin-1 is a transmembrane receptor belonging to the immunoglobulin superfamily. It shares 50% amino-acid identity with the human tumor suppressor molecule deleted in colon cancer (DCC) [9], and is a netrin receptor-like DCC that binds repulsive guidance molecules (RGM); its functions as a RGM receptor have been studied [10-11]. While DCC expression is restricted to the nervous system, neogenin-1, a cell adhesion molecule, is widely expressed in a variety of developing tissues, such as in the gastrointestinal tract of chickens, as well as on the surface of developing neurons in the heart, lungs, and pancreas [12]. Moreover, due to its similarity to DCC, neogenin-1 was thought to mediate axon guidance in a netrin-dependent manner. However, the knockout of the neogenin-1 gene in mice did not affect axon guidance phenotypes [13], and its DCC-independent functions have been determined, including the regulation of bone formation, mammary gland morphogenesis, and skeletal myofiber development [14-16].

Neogenin-1 seems to be involved in tissue growth regulation, cell to cell recognition, cellular transition from proliferation to terminal differentiation, and cell migration. Although neogenin-1 expression has been detected in many adult tissues, there is no information available regarding the status of neogenin-1 expression in the human ovary. Neogenin-1 is present in tissues where active growth takes place, and overexpression of neogenin-1 has been observed in a wide variety of human cancers including those of the breast, pancreas, cervix, colon, medulloblastoma and rectum [17-19]. Previous studies have also shown that neogenin-1 may promote cell migration and cell-cell adhesion in human vascular smooth muscle in addition to zebrafish neurectoderm [9, 20]. Also, neogenin-1 is involved in various aspects of cancer progression and metastasis [9]. It is expressed and has a role in colon cancer [21], breast cancer [22], pancreatic cancer [23], glioma [24], and esophageal squamous cell carcinoma (ESCC) [25]. Interestingly, its role and expression levels vary significantly among cancers affecting different organs. For example, low expression of neogenin-1 is seen in breast cancers, colon cancers and glioma, moderate expression in pancreatic cancer and high expression in ESCC compared to the same healthy organs. However, the role of neogenin-1 remains unclear because the opposite functions on cancer have been reported in several cancers [9, 22, 24].

In this study, we first determined the expression of neogenin-1 in gastric cancer patients and gastric cancer cells. The function of neogenin-1 was also determined by assessment of gain and loss of function of neogenin-1 in gastric cancer cell proliferation and migration. Moreover, we also determined that neogenin-1 expression is regulated by transcriptional factor HSF-1, mediated stress response and the activity of specific heat shock proteins (HSPs) [26-28]. HSF-1 expression is elevated in various cancers, including human prostate carcinoma cell lines, and has been shown to support proliferation of several cancer cell lines [27-31]. Finally, we assessed how galectin-3 regulates the expression of neogenin-1, specifically through interaction with HSF-1. Therefore, we demonstrate herein the effects of neogenin-1 on gastric cancer cell proliferation and migration, as well as the regulation of its expression by HSF-1 and galectin-3 interactions.

## RESULTS

### Detection of expression level of neogenin-1 in tissues of gastric cancer patients

**Figure 1:**
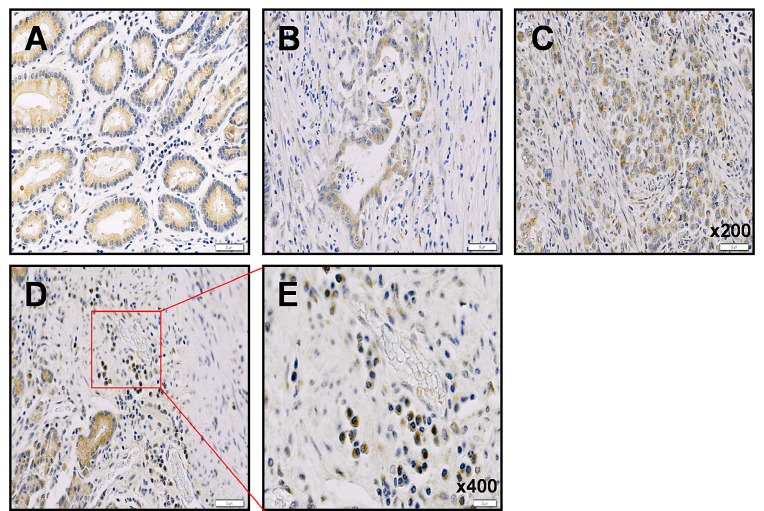
Immunohistochemical analysis of neogenin-1 expression in human gastric cancer patient tissues The evaluation of neogenin-1 expression investigated in gastric cancers. **(A)** Adjacent normal tissues of gastric cancer, **(B)** Intestinal type adenocarcinoma gastric cancer tissue, **(C)** Diffuse type adenocarcinoma gastric cancer tissue, **(D and E)** signet ring cell carcinoma gastric cancer, on tissue microarrays by immunohistochemical method. Neogenin-1 (brown) is mostly expressed in cytoplasms of each tissue through staining intensities. **(A-D)** panels magnification, x 200, **(E)** panel magnification, x400

We investigated the expression of neogenin-1 in 59 cases of gastric cancer tissues and normal tissues of gastric cancer patients (Fig. 1). The expression of neogenin-1 was detected in normal tissues, especially in cytoplasm of glandular cells (Fig. 1A). Although the case number of patients was not enough, we found the expression levels of neogenin-1 was higher in diffuse type than intestinal type of gastric cancer tissues (Fig. 1B-C). Interestingly, neogenin-1 was highly detected in signet ring cell carcinoma of diffuse type gastric cancer tissues (Fig. 1D-E), which has a metastatic property on the various cancer types [32-34].

### Effect of the ablation of neogenin-1 or netrin-1 on gastric cancer cell proliferation and migration

**Figure 2:**
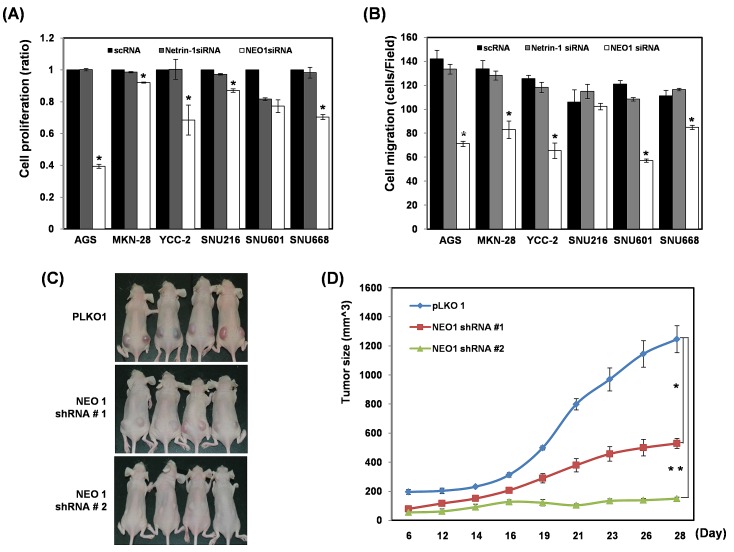
Effect of each ablation of neogenin-1 or netrin-1 on the proliferation and migration of six gastric cancer cells and on AGS xenografted mice **(A)** Cell proliferation was checked in 6 gastric cancer cell lines (AGS, MKN-28, YCC-2, SNU-216, SNU-601, and SNU-668) after transfection with scRNAs, neogenin-1 siRNA or netrin-1 siRNA (* *p***<0.001). (B)** Six gastric cancer cell lines were transfected with scRNAs, neogenin-1 siRNA or netrin-1 siRNA, and analyzed by migration and invasion assays, with the results presented as histograms (* *p*<0.04). All experiments were performed in triplicate. **(C)** The effect of neogenin-1 on tumor growth of xenografts in nude mice. 50ul of AGS SQ cells (1 × 10^6^) with Matrigel were implanted into Balb/c-nude mice to form subcutaneous xenografts. Tumor volumes were measured at different time points and are presented in photographs taken at day 28 and in a graph **(D)**. Data are presented as the mean and standard error of the mean (SEM). Unpaired student's t-test was used for the comparison between the two groups (*, ** *p*<0.002).

Whereas neogenin-1 expression was highly detected in 10 of 12 gastric cancer cell lines, both ligand of neogenin-1, netrin-1 and RGMa were low expressed or unexpressed in most gastric cancer cell lines, respectively (Suppl. Fig. 1). We depleted the expression of neogenin-1 and netrin-1 by each siRNA to determine the effect on cell proliferation in six gastric cancer cell lines (Suppl. Fig. 2 and Fig. 2A). The cell proliferation was reduced by the ablation of neogenin-1, and the strongest reduction was detected in AGS cells and YCC-2 cells. However, the ablation of netrin-1 did not show any visible reduction of proliferation in six gastric cancer cell lines. The cell migration by ablation of neogenin-1 distinctively lessened the migration of 5 of the gastric cancer cells except SNU-216, whereas the ablation of netrin-1 had no significant reduction (Fig. 2B). Our results suppose that the effect of neogenin-1 on the proliferation and migration gastric cancer cells and is independent of its ligand netrin-1.

### Effect of ablation of neogenin-1 *in vivo* in gastric cancer cell xenografted mice

We prepared two clones of neogenin-1 shRNA stable expressing AGS cells and inoculated in nude mice (Fig. 2C-D). Compared to tumors growth made from negative control LacZ shRNA expressing AGS cells, the neogenin-1 ablation by both shRNAs showed reduced tumor growth in xenografted mice. These data strongly suggest that neogenin-1 accelerates *in vivo* gastric cancer progression.

### Transient transfection of neogenin-1 accelerated the proliferation, migration and invasion of SNU-668 gastric cancer cells

**Figure 3:**
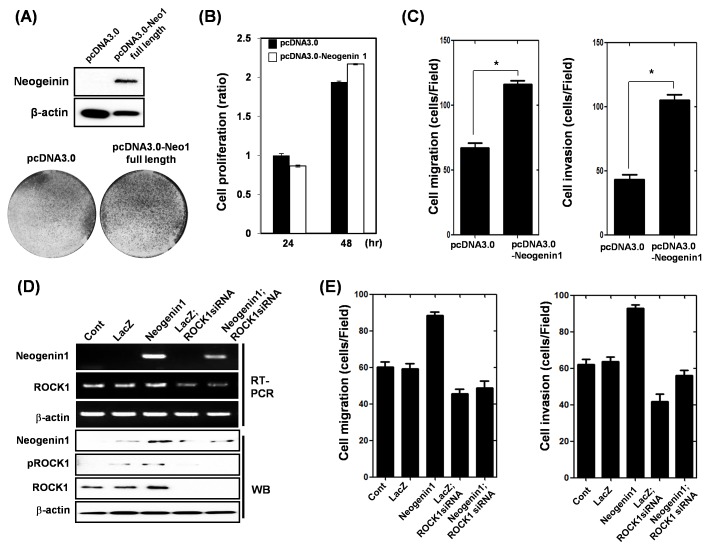
Effect of transient transfection of neogenin-1 in SNU-668 gastric cancer cells on the proliferation, migration and invasion **(A-C)** SNU-668 cells were transfected with pcDNA3.0 control vector and pcDNA3.0-neogenin-1 expression vector for 48 h. **(A)** Western blot analysis of neogenin-1 protein expression and crystal violet staining of cells, **(B)** Cell proliferation measured by WST assay (* *p*<0.001), **(C)** Cell migration assay and cell invasion assay (* *p*<0.0001), were performed as described in the methods. **(D)** Expression levels of neogenin-1 and ROCK1-1 were detected by RT-PCR and Western blot analysis in SNU-668 cells, which were infected with lentivirus-containing LacZ or neogenin-1 full-length, and then were transfected with scRNA or ROCK1 siRNA. β-actin was used as a loading control. **(E)** The cells were analyzed with cell migration and invasion assays, and data are presented as histograms (*, ** *p*<0.001). All experiments were performed in triplicate.

Neogenin-1 over-expression plasmids were transfected into SNU-668 cells, which demonstrate low expression of neogenin-1 in twelve gastric cancer cell lines (Fig. 3A-C). In all cases, cell proliferation, migration and invasion were increased by neogenin-1 over-expression. Previously, it was reported that neogenin-1 regulates Rho/Rac/ROCK1 activity [35]. We reduced neogenin-1 expression, and then detected an associated decrease in both phosphorylated ROCK1 and total ROCK1 protein levels in AGS cells (Suppl. Fig. 3A). The depletion of ROCK1 also reduced AGS cell migration and invasion (Suppl. Fig. 3B). To establish whether neogenin-1 induced gastric cancer cell migration and invasion through ROCK1 activation, we over-expressed neogenin-1 in SNU-668 cells and decreased the expression of ROCK1 by ROCK1 siRNA. Then, cell migration and invasion assays were performed using these cells (Fig. 3D-E). As shown in Figure 3E, over-expression of neogenin-1 increased cell migration and invasion, whereas ROCK1 depletion reversed these effects. Moreover, the reduction in phosphorylation of ROCK1 was also detected in *in vivo* neogenin-1-depleted tumors of AGS cell-xenografted mice (Suppl. Fig. 4). Taken together, neogenin-1 appears to regulate ROCK1 activation leading to gastric cancer cell migration and invasion.

### Ablation of galectin-3 and neogenin-1 inhibits AGS gastric cancer cell migration and invasion

**Figure 4:**
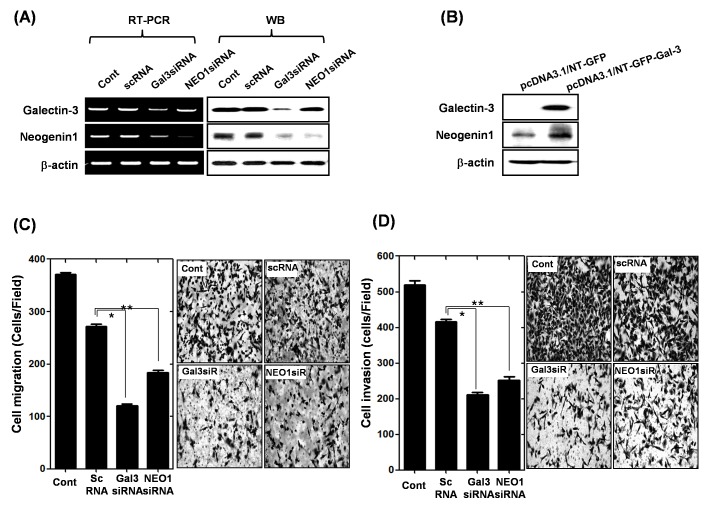
Effect of galectin-3 depletion on the expression of neogenin-1 in human gastric cancer cells **(A)** mRNA and protein levels of galectin-3 and neogenin-1 after transfection of AGS cells with scrambled siRNA (scRNA), galectin-3 siRNA, or neogenin-1 siRNA. β-actin was used as a loading control. **(B)** Expression levels of galectin-3 and neogenin-1 in SNU-638 cells as determined by western blotting after transfection with pcDNA3.1/NT-GFP-galectin-3 and vector control of pcDNA3.1/NT-GFP. **(C-D)** After transfection of AGS cells with scrambled siRNA (scRNA), galectin-3 siRNA, or neogenin-1 siRNA, the migration and invasion assay were performed as described in “Methods” and quantification of **(C)** cell migration (*, ** *p*<0.0001) and **(D)** cell invasion are presented as a histogram (*, ** *p*<0.0001). All experiments were performed in triplicate.

In our previous microarray study, we found galectin-3 regulates neogenin-1 expression (GSE 29630), so we focus on the galectin-3 and neogenin-1 relationship. The expression levels of galectin-3 and neogenin-1 were checked in 12 gastric cancer cell lines (Suppl. Fig. 1), and AGS (high expression of galectin-3) and SUN-638 (without expression of galectin-3) were selected for the following experiments. First, we transfected AGS cells with galectin-3 siRNA or neogenin-1 siRNA, and detected the expressions of galectin-3 and neogenin-1 using RT-PCR and Western blotting, respectively (Fig. 4A). Ablation of galectin-3 could reduce the expression of neogenin-1, but neogenin-1 had no effect on galectin-3 expression. After we over-expressed galectin-3 in SNU-638 cells, we detected an increase in neogenin-1 expression (Fig. 4B). Taken together, these results suggest that galectin-3 regulates neogenin-1 expression. To determine the effect of neogenin-1 on cell migration and invasion, we performed cell migration and invasion assays after treatment with each siRNA of galectin-3 and neogenin-1 in AGS cells (Fig. 4C-D). The galectin-3 or neogenin-1-depleted cells were no longer mobile, and the total number of migrated cells and invasive cells was reduced by almost half.

### Galectin-3 promotes motility of human gastric cancer cells through up-regulation of neogenin-1 expression

We also examined whether galectin-3 regulates cell motility and invasion through neogenin-1 expression. Galectin-3 overexpressing cells by a galectin-3-containing lentivirus construct showed an increased level of neogenin-1 protein, and we abolished this increase using neogenin-1 siRNA (Suppl. Fig. 5A). Interestingly, galectin-3 over-expression also induced phosphorylation of ROCK1, whereas ablation of neogenin-1 reduced its phosphorylation (Suppl. Fig. 6A). Cell migration and invasion assays were also performed on these cells. Galectin-3 overexpression increased cell migration and invasion, while neogenin-1 silencing significantly reduced the increases caused by galectin-3 overexpression (Suppl. Fig. 5B). These results suggest that galectin-3 promotes gastric cancer cell motility by increasing neogenin-1 expression.

### Transcriptional regulation of neogenin-1 by galectin-3 through interaction with HSF-1 transcriptional factor

**Figure 5:**
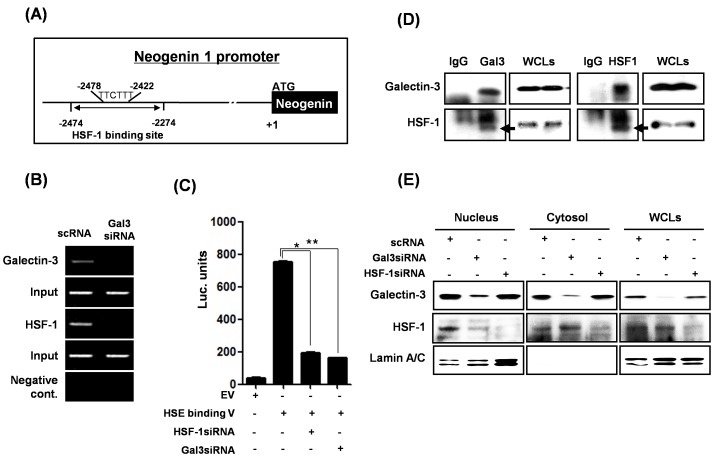
Transcriptional regulation of neogenin-1 through interaction with galectin-3 and HSF-1 transcriptional factor **(A)** The proximal HSF-1 binding site is located between -1478 and -1470 from the initiation codon of *NEO1* gene. **(B)** Chromatin immunoprecipitation assay was performed as described in “Methods.” Total genomic DNA was used in the input lane as a control for the PCR assay. **(C)** Luciferase activity of HSF-1 in AGS cells treated with galectin-3 siRNA or HSF-1 siRNA. A luciferase assay was performed using a HSF-1 binding site luciferase vector transfection with galectin-3 siRNA or HSF-1 siRNA (*, ** p<0.001). The experiments were performed in triplicate. **(D)** The detection of the interaction between galectin-3 and HSF-1 by immunoprecipitation was performed as described in “Methods,” and then galectin-3 and HSF-1 were detected by western blot analysis. Whole-cell lysate (WCL) was used as a loading control. **(E)** Protein levels of galectin-3 and HSF-1 were detected in the nuclear and cytosol fractions of AGS cells which were transfected with galectin-3 or HSF-1 siRNA. Lamin A/C was used as a control for the nuclear fraction.

In order to further explore how galectin-3 increases neogenin-1 expression in gastric cancer cells, we focused on the transcription factor, heat shock factor 1 (HSF-1), as a major regulator of heat shock protein transcription. We analyzed the binding site of HSF-1 in the neogenin-1 promoter (Fig. 5A), and examined the DNA binding activity of HSF-1 with or without galectin-3 by ChIP assay (Fig. 5B). In the presence of galectin-3, HSF-1 bound to the promoter regions of neogenin-1, but not in the absence of galectin-3. A HSF-1 luciferase assay was also performed to detect transcriptional activity of HSF-1 with or without galectin-3 expression (Fig. 5C). Transcriptional activity of HSF-1 was decreased in the absence of galectin-3. Therefore, we carried out an immunoprecipitation assay, and observed a strong interaction between galectin-3 and HSF-1 (Fig. 5D). Interestingly, the localization of HSF-1 was changed by galectin-3 (Fig. 5E). We separated the cytosol and nuclear fractions of cell lysates treated with galectin-3 siRNA or HSF-1 siRNA. In the absence of galectin-3, most HSF-1 expression was detected in the cytosol, suggesting less transcriptional activity. However, the absence of HSF-1 had no effect on the galectin-3 localization. These results suggest that galectin-3 contributes to the binding of the neogenin-1 promoter by interacting with HSF-1 protein, thereby increasing neogenin-1 expression by transcriptional regulation.

### HSF-1 promotes motility of human gastric cancer cells through up-regulation of neogenin-1 expression

**Figure 6:**
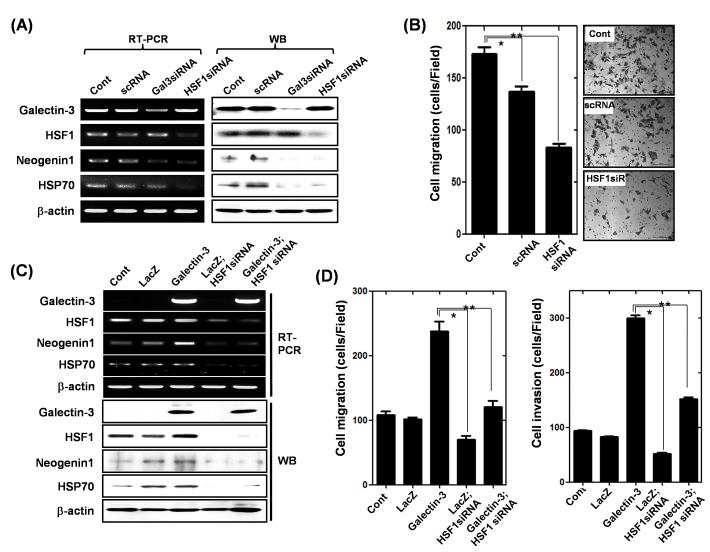
Effect of HSF-1 depletion on the expression of neogenin-1 and gastric cancer cell motility **(A)** Detection of mRNA and protein levels of galectin-3, HSF-1, HSP70, and neogenin-1 after transfection of AGS cells with scRNA, galectin-3 siRNA and HSF-1 siRNA. β-actin was used as a loading control. **(B)** After transfection of AGS cells with scrambled siRNA (scRNA), or HSF-1 siRNA, the migration assay were performed and quantification of cell migration was presented as a histogram (*, ** *p*<0.001). **(C)** Expression levels of galectin-3, HSF-1, HSP70 and neogenin-1 were detected by RT-PCR and Western blotting analysis in SNU-638 cells, which were infected with lentivirus-containing LacZ or galectin-3 full-length, and then followed by transfection with HSF-1 siRNA or scRNA. β-actin was used as a loading control. **(D)** The cells were analyzed by migration assay and invasion assay, and data are presented as histograms (*, ** *p*<0.001). All experiments were performed in triplicate.

Because HSF-1 increased neogenin-1 expression, we determined the regulatory effect of HSF-1 on migration of gastric cancer cells (Fig. 6). First, we examined the decreased expression of both neogenin-1 and HSP70 [36], a downstream target of HSF-1, after the depletion of HSF-1 or galectin-3 (Fig. 6A). The ablation of HSF-1 reduced the gastric cancer cell migration significantly (Fig. 6B). Moreover, increased expression of neogenin-1 by galectin-3 over-expression was reduced in the absence of HSF-1. HSP70 expression was also increased by galectin-3 over-expression, and was diminished in the absence of HSF-1 (Fig. 6C). The increased cell migration and invasion by galectin-3 overexpression decreased with HSF-1 depletion (Fig. 6D). Taken together, our results demonstrated that HSF-1 increases gastric cancer cell motility through regulation of neogenin-1 expression, and the transcriptional activation of HSF-1 is regulated by galectin-3.

### Correlation between neogenin-1 and galectin-3 expression in malignant and normal tissues from gastric cancer patients

**Figure 7:**
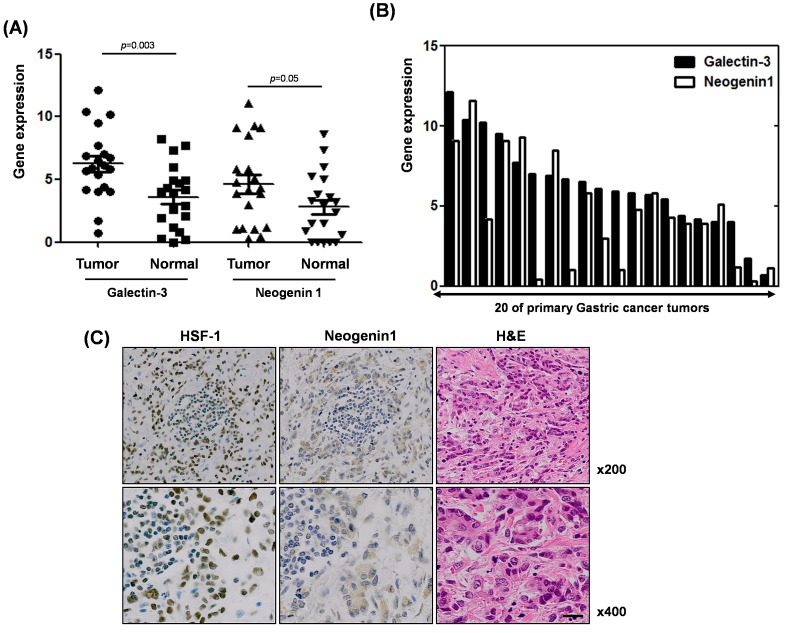
Positive association between galectin-3 and neogenin-1 expression, and HSF-1 and neogein1 expression in malignant and normal tissues from gastric cancer patients **(A)** mRNA expression levels of galectin-3 and neogenin-1 from malignant tissues of 20 gastric cancer patients are presented in a histogram. **(B)** Expression levels of galectin-3 and neogenin-1 were calculated using ImageJ in normal and tumor tissues of 20 gastric cancer patients. Those *p* values were calculated using student T-test. **(C)** Protein expression of HSF-1 and neogenin-1 in the malignant tissues of gastric cancer patients were shown by immunohistochemical staining (brown) with hematoxylin and eosin (H&E) and detected by inverted light microscope. Magnification; X200 (up) and X400 (down).

The expression levels of galectin-3 and neogenin-1 were analyzed in 20 gastric cancer patients obtained from the National Cancer Center of Korea. Total RNA of normal and cancer tissues from these patients were prepared and RT-PCR was performed (Suppl. Fig. 6). We quantified galectin-3 and neogenin-1 expression using ImageJ and created a graph (Fig. 7A). The expression levels of both galectin-3 and neogenin-1 were higher in cancer tissues than in normal tissues. Furthermore, analysis of 20 primary human gastric tumors revealed an overall positive correlation between increased active expression of galectin-3 and elevated neogenin-1 expression, except in 6 tissues (Fig. 7B). We could not determine clinical implications from these samples, maybe due to the small number of samples. However, these results revealed a significant positive correlation between the expression levels of galectin-3 and neogenin-1 in gastric cancer patient tissues.

Moreover, we also established the parallel protein expression and co-localization of HSF-1 and neogenin-1 in malignant tissues of gastric cancer patients (Fig. 7C). Therefore, our results strongly suggest that neogenin-1 was more detected in malignant tissues and its expression was positively correlated with both HSF-1 and galectin-3 in the malignant tissues of gastric cancer patients.

## DISCUSSION

In this study, we confirmed for the first time that neogenin-1 is highly expressed in gastric cancer and promotes gastric cancer proliferation and migration. It is unclear why neogenin-1 is differentially expressed across organs. For example, the loss of expression of neogenin-1 in breast cancer and colorectal cancer has already been studied, and it appears that the RGMa/neogenin-1 interaction inhibits cancer growth and migration [21-22]. In our study, the expression of neogenin-1 was relatively high and easily detectable in twelve gastric cancer cell lines. In human tissues of gastric cancer patients, the expression level was highly detected in malignant tissues than normal counterparts, especially in diffuse type of signet ring cell carcinomas. It may provide the cue that neogenin-1 is involved in motility of gastric cancer cells, because signet ring cell carcinoma has a metastatic property on the various cancer types [32-34]. Neogenin-1 ligands netrin-1 and RGMa were expressed at low levels or not at all in gastric cancer cells, respectively. Moreover, only ablation of neogenin-1 reduced gastric cancer cell proliferation and migration, whereas ablation of netrin-1 had little effect. Neogenin-1 is thought to be up-regulated during gastric cancer development and is involved in gastric cancer growth and metastasis in a ligand-independent manner. In contrast to DCC, neogenin-1 appears to have varying expression and function depending on the organ from which the cancer originated [9]. We suggest that alternatively spliced isoforms of neogenin-1 are expressed in various developmental tissues and development stages, and that these isoforms may function differently. However, the regulatory mechanisms for these spliced isoforms remain to be determined. Moreover, detailed analyses of the expression and function of DCC and neogenin-1 are also needed at the single-cell level as well as in different organs.

To understand how neogenin-1 expression is up-regulated in gastric cancer, we searched for transcription factor binding sites in the neogenin-1 promoter region using a transcription factor binding site prediction program. Next, we verified that the HSF-1 transcriptional factor interacts with the neogenin-1 promoter. Many cancers histologically exhibit activated HSF-1 and increased HSP levels, and activate oncogenes to regulate cell proliferation and cell motility [37-38]. Another study also demonstrated that HSF-1 plays an essential role in the development of lymphoma in p53-deficient mice, and of carcinomas in the Ras tumor model [28, 39]. Whether the multifaceted HSF-1-mediated stress response plays a causal, supportive, or inhibitory role in mammalian oncogenesis remains unclear. Interestingly, we first demonstrated that HSF-1 activation is induced by galectin-3 interaction. Usually HSF-1 is activated by extracellular stresses, e.g., heat shock, cytokines or ROS [40]. Over-expression of galectin-3 facilitated the nuclear-localization of HSF-1 and binding to the promoter of neogenin-1. Related to this, HSF-1-mediated cell motility was caused by an increase in neogenin-1. Moreover, parallel and high expression of neogenin-1 and galectin-3, as well as neogenin-1 and HSF-1, was detected in human gastric cancer tissue. These data strongly suggest that over-expression of HSF-1 and galectin-3 jointly increases neogenin-1 expression both *in vivo* and *in vitro*. Neogenin-1 also appears to regulate gastric cancer cell motility through the activation of ROCK [35]. The function of ROCK1 is cell type-specific and remains controversial. However, there are several studies demonstrating that up-regulation of ROCK signaling contribute to cancer progression and metastatic behavior [41-42]. Moreover, ROCK1 increases gastric cancer cell migration and invasion [43], and the inhibition of ROCK1 induces apoptosis in gastric cancer cells [44], suggesting that neogenin-1 increases the activation of ROCK1 and, in turn, promotes gastric cancer cell survival and motility. Taken together, our results suggest that neogenin-1 increases gastric cancer cell motility, and its expression is highly regulated in gastric cancers through interactions with galectin-3 and HSF-1. Further study will be critical in determining the role and regulation of neogenin-1 in various cancer types, as well as the roles of neogenin-1, galectin-3 and HSF-1 regulation in cancer metastasis.

## METHODS

### Cell culture

12 human gastric cancer cell lines (AGS, MKN28, YCC-2, KATOIII, SNU-1, SNU-5, SNU-16, SNU-216, SNU-601, SNU-638, SNU-668, and SNU-719) obtained from Korea Cell Line Bank were cultured in DMEM or RPMI 1640 medium supplemented with 5% fetal bovine serum (FBS) and 1% Antibiotics as described previously [45]. The KCLB authenticate the phenotypes of these cell lines.

### siRNA transfection

Galectin-3, neogenin-1, netrin-1, and HSF-1 siRNA transfections were performed with Lipofectamine RNAiMAX reagent (Invitrogen, USA) following the manufacturer's instructions. The coding strand of human galectin-3 siRNAs was 5'-AUAUGAAG CACUG GUGAGGUCUAUG-3' and that of human neogenin-1 siRNA was 5'-AGAUCUGGAGGUU UCACAUCUUUGG-3', and each was purchased from Invitrogen. Human netrin-1 siRNA was 5'-GGGUGCCCUUCCAGUUCTA-3' and human HSF-1 siRNA was 5'-GCGGCAGC UCAACAUGUAU-3', and each was purchased from Genolution (Korea).

### RNA isolation and reverse transcription-polymerase chain reaction (RT-PCR)

Total RNA was extracted from human gastric cancer cells and gastric cancer patient tissues with TRIzol reagent (Invitrogen, USA) according to the manufacturer's protocol. RT was carried out using a RT system (Promega, USA), and PCR was performed with Ex-taq DNA polymerase (TaKaRa, Japan). The sequences of primers were as follows: Galectin-3: 5'-CAGTGCTCCTGGAGGCTATC-3' (sense) and 5'-AAGGGGAAGGCTGACTGTCT-3' (anti-sense); *NEO1*: 5'- AGGAGAGATGCAAGTAACCA -3' (sense) and 5'-GTTTCCCACGTAACAGTGAT-3' (anti-sense); NTN-1 (Netrin-1); 5'- TGCAAGTGTCCCAAAATCAA-3' (sense) and 5'-GCACTTGCCCTTCTTCTCAC-3' (anti-sense); HSP70; 5'- AGAGCCGA GCCGACAGAG -3' (sense) and 5'-CACCTTGCCGTGTTGGAA-3' (anti-sense) β-actin: 5'-AGCCTCGCCTTTGCCGA-3' (sense) and 5'-CTGGTGCCTGGGGCG-3' (anti-sense).

### Fractionation of cellular extracts and Western blot analysis

Nuclear or cytoplasmic extracts were prepared from AGS cells after galectin-3 siRNA and HSF-1 siRNA treatments. These experiments have been described in a previous study [46]. Western blot analysis was carried out using the methodology from a previous study [47]. Briefly, cells were lysed in RIPA buffer (Biosesang, Korea) containing a protease inhibitor cocktail (Sigma, USA). The primary antibodies were: anti-β-actin, anti-galectin-3, anti-neogenin-1, anti-HSF-1, and anti-HSP70 from Santa Cruz, and anti-ROCK-1 from Cell Signaling, also, anti-pROCK-1(T455/S456) antibody from Bioss. Proteins of interest were detected using ECL solutions (Amersham Life Science) with an LAS-3000 (Fujifilm) detector according to the manufacturer's directions.

### Immunoprecipitation assays

Cell lysates containing 750 μg proteins was pre-cleared by incubation with 40 μl protein-A/G linked agarose beads (Santa Cruz, CA, USA) for 1 hour at 4°C. After the beads were spun down, the supernatant was incubated with 1 μg its specific antibody (anti-galectin-3 and anti-HSF-1) overnight at 4°C, followed by incubation with 40 μl protein-A/G agarose beads for 1 hour. Mouse IgG (Santa Cruz, CA, USA) was used as the negative control. After the incubation, beads were washed three times in a RIPA buffer before being dissolved in a SDS-PAGE loading buffer. Western blot analysis was performed as described above.

### Transfilter migration and invasion assays

Briefly, AGS, MKN-28, YCC-2, SNU-216, SNU-601, SNU-668 and SNU-638 cells were transfected with galectin-3, neogenin-1, netrin-1, and ROCK1 siRNAs. One day after the transfection, cells (AGS-1×10^4^, MKN-28-1×10^4^, YCC-2-1×10^4^, SNU-216-1×10^4^, SNU-601-1×10^4^, SNU-668-1×10^4^ and SNU638-1×10^4^ each well) were isolated and added to upper Transwell (Corning Costar, USA) chambers with 0.5 mg/ml collagen type I (BD bioscience, Korea)-coated filters for migration assay, and with 1/15 dilution of Matrigel (BD bioscience, Korea)-coated filters for invasion assays. RPMI 1640 containing 10% FBS and 1% antibiotics was added to the lower chamber and incubation was continued for 20 hours. Cells that migrated or invaded to the lower chamber were quantified after H&E staining. For quantification, cells were counted at 5 randomly selected areas in each well using wide-field microscopy. Data were expressed as mean ± SD from three independent experiments.

### Galectin-3 over-expression construction and infection

The galectin-3 overexpression vector (pcDNA3.1/NT-GFP-galectin-3) and lentiviral vectors containing galectin-3 over-expression were constructed by inserting the galectin-3 gene into the lentiviral vector pLL3.7, as described in a previous study [48]. The plasmid pcDNA3.0–neogenin-1 kindly provided by Dr. Patrick Mehlen (Université de Lyon), was transiently transfected into cells using Lipofectamine 2000 reagent (Invitrogen) according to the manufacturer's instructions.

### Crystal violet staining

Cells were transfected with 4ug of pcDNA3.0 Eempty vector and pcDNA3.0-Neogenin-1 full length by reverse transfection method using Lipofectamine 2000. For crystal violet staining, cells were plated into 6 well culture dishes and stained with crystal violet 2 days after transfection [49].

### Cell proliferation analysis

Inhibition of cell proliferation by netrin-1, neogenin-1 siRNA treatment was measured by WST assay. The AGS, MKN-28, YCC-2, SNU-216, SNU601 and SNU668 cells were plated in 96-well culture plates (3 × 10^3^ per well). After incubation for 24 h, the cells were treated with 20nM of netrin-1, neogenin-1 siRNA treatment for 48 hr. WST solution (Daeil, korea) was subsequently added to each well. After 1-3h of additional incubation, the plate was shaken gently. The absorbance was measured on an ELISA reader at a test wavelength of 450 nm.

### Chromatin immunoprecipitation assay

We performed a chromatin immunoprecipitation (ChIP) assay using a ChIP assay kit (Upstate). Samples were applied to dishes after galectin-3 siRNA treatment and assays were conducted following the manufacturer's instructions. Anti-galectin-3, and anti-HSF-1 and normal mouse/rabbit IgG were used to immunoprecipitate DNA-containing complexes. Prior to PCR, primers were prepared for HSF-1 with neogenin-1 promoter binding sites, neogenin-1 promoter (-2474) 5'-CCTAGAGCAGGTGGCTTCTG-3' and (-2274) 5'- GAATCCCAGTG TCCGAGGT-3'. PCR was performed with Ex taq (Takara).

### Luciferase activity of HSF-1 promoter

For HSF-1 reporter assays, a HSF-1 binding site luciferase construct (pGL3-HSE, containing 3 copies of HSE third pentamer consensus sequence reporter plasmid [50] was used. Gastric cancer cells were transfected with pGL3-HSE and a β-gal control construct, and HSF-1 promoter activities were analyzed by luciferase gene activity as described previously [51].

### Preparation of Neogenin-1-depleted AGS gastric cancer cell xenografted mice

All animal experiments were approved by the Institutional Review Board of the National Cancer Center (NCC Korea) and performed in specific pathogen-free facilities and under conditions in accordance with the Guidelines for the Care and Use of Laboratory Animals of NCC (NCC-11-034D). The preparation of xenografted mice has been described in a previous study [52].

### Fractionation of cellular extracts

Nuclear or cytoplasmic extracts were prepared from AGS cells after galectin-3 siRNA and HSF-1 siRNA treatments. These experiments have been described in a previous study [5].

### Human gastric cancer tissue micro-array analyses and Preparation of frozen tissues from gastric cancer patients

Core tissue biopsy specimens (2-mm diameter) were obtained from individual paraffin-embedded gastric carcinomas (donor blocks) and arranged in new recipient paraffin blocks (tissue array blocks) using a trephine apparatus (Superbiochips Laboratories, Seoul, Korea). Immunohistochemical analysis of HSF-1 and Neogeinin1 was performed as described previously [53]. Two pairs of 2-mm-sized biopsy specimens were obtained from 20 patients with gastric adenocarcinoma during diagnostic endoscopic submucosal dissection. Immediately after biopsy, these tissue samples were frozen in liquid nitrogen in a deep freezer at −70°C until experimental use. All participants provided written informed consent. All experiments were approved by the Institutional Review Board of the National Cancer Center as approval number NCCNSH 03-024.

### Statistics analysis

All data were obtained from at least three independent experiments and are presented as mean ± SD, unless otherwise indicated. Statistical analysis was performed using one-way ANOVA and student T-test methods. Data were considered significant if *p*<0.05.

## SUPPLEMENTARY FIGURES


