# Supplementary material for: Up-regulation of neogenin-1 increases cell proliferation and motility in gastric cancer
Source: Oncotarget. 2014 May 13;5(10):3386–98. doi: 10.18632/oncotarget.1960 (PMC4102817; doi:10.18632/oncotarget.1960)
Supplement: Supplementary file 1 [file oncotarget-05-3386-s001.pdf]

Up-Regulation of Neogenin-1 Increases Cell Proliferation and Motility in Gastric Cancer

Supplementary Material

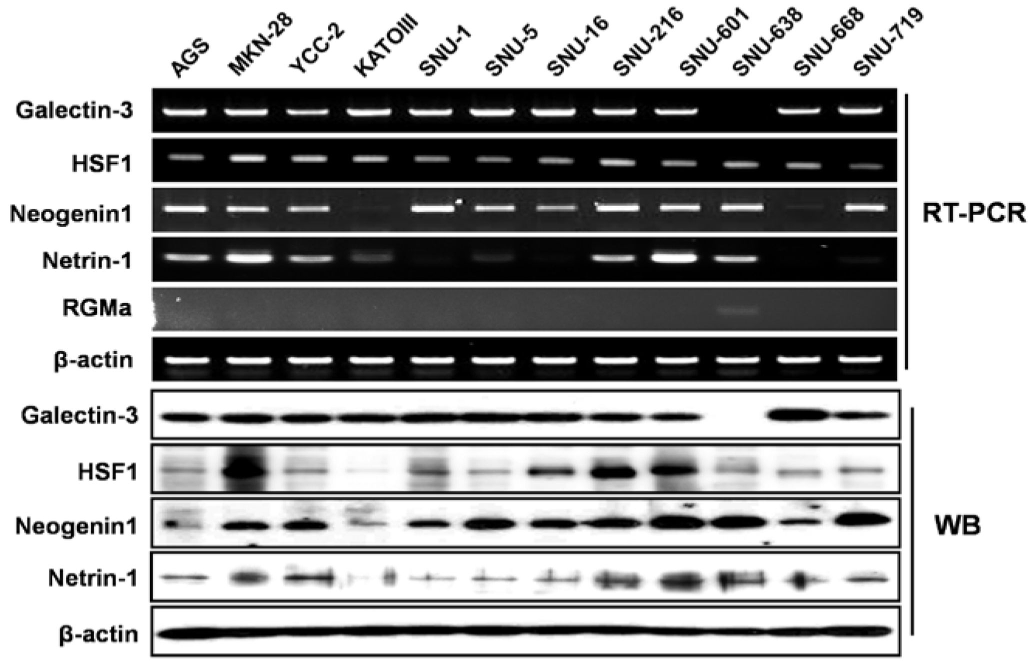

Supplementary Figure 1: Expression levels in 12 gastric cancer cell lines. (A) mRNA and protein expression levels of neogenin-1, netrin-1, RGMa, and galectin-3 were determined by RT-PCR and western blotting, respectively, in 12 gastric cancer cell lines: AGS, MKN28 (gastric epithelial cells), YCC-2, KATOIII (derived from metastatic site: pleural effusion; supraclavicular and axillary lymph nodes and Douglas cul-de-sac), SNU-1, SNU-5, SNU-16, SNU-216, SNU-601, SNU-638, SNU-668, and SNU-719 (gastric cancer cells from Seoul National University). β-actin was used as a loading control.

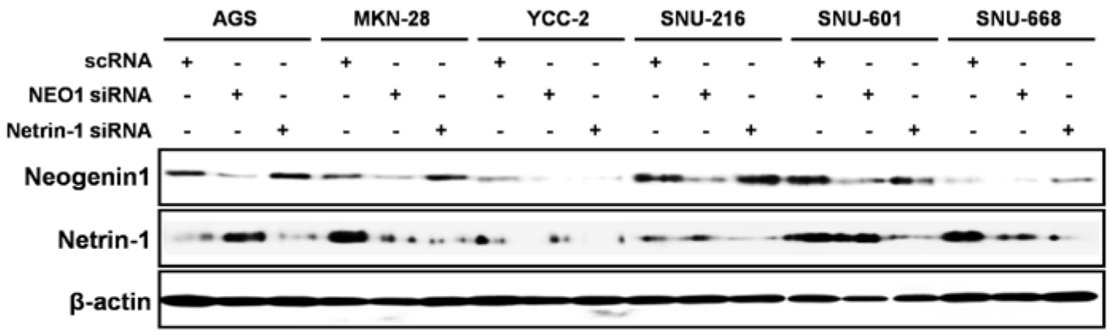

Supplementary Figure 2: The detection of protein expression levels by western blotting after neogenin-1 siRNA or netrin-1 siRNA transfection. After siRNA transfection with each neogenin-1 or netrin-1 in 6 kinds of gastric cancer cell lines, and the expression levels of neogenin-1 or netrin-1 were detected by western blotting. β-actin was used as a loading control.

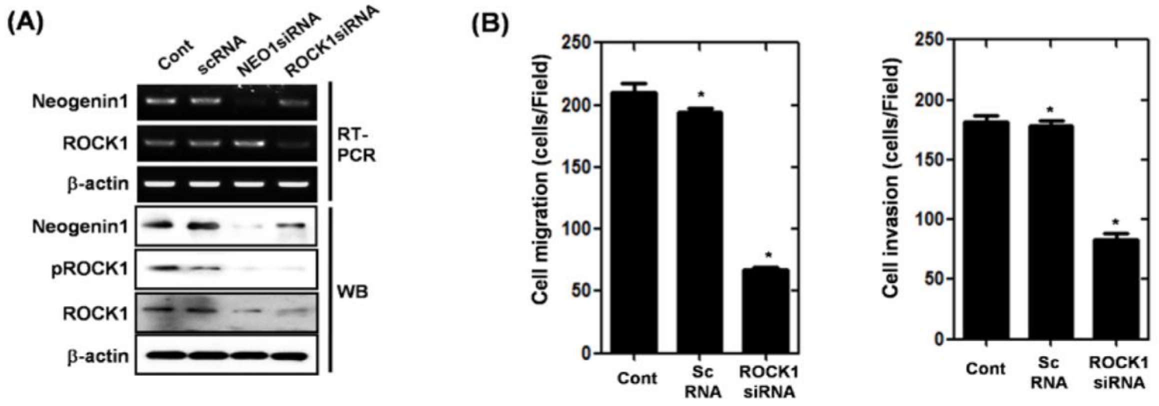

Supplementary Figure 3: Effect of neogenin-1 depletion on ROCK1 phosphorylation and cell migration and invasion. (A) Expression levels of neogenin-1, ROCK1 and pROCK1 were detected by RT-PCR and western blotting analysis in AGS cells, which were transfected with neogenin-1 and ROCK1 siRNA or scRNA as a negative control. β-actin was used as a loading control. **(B)** These cells were analyzed by migration assay and invasion assay, and results are presented as histograms (* $p<0.001$ vs. Cont; one-way ANOVA). All experiments were performed in triplicate.

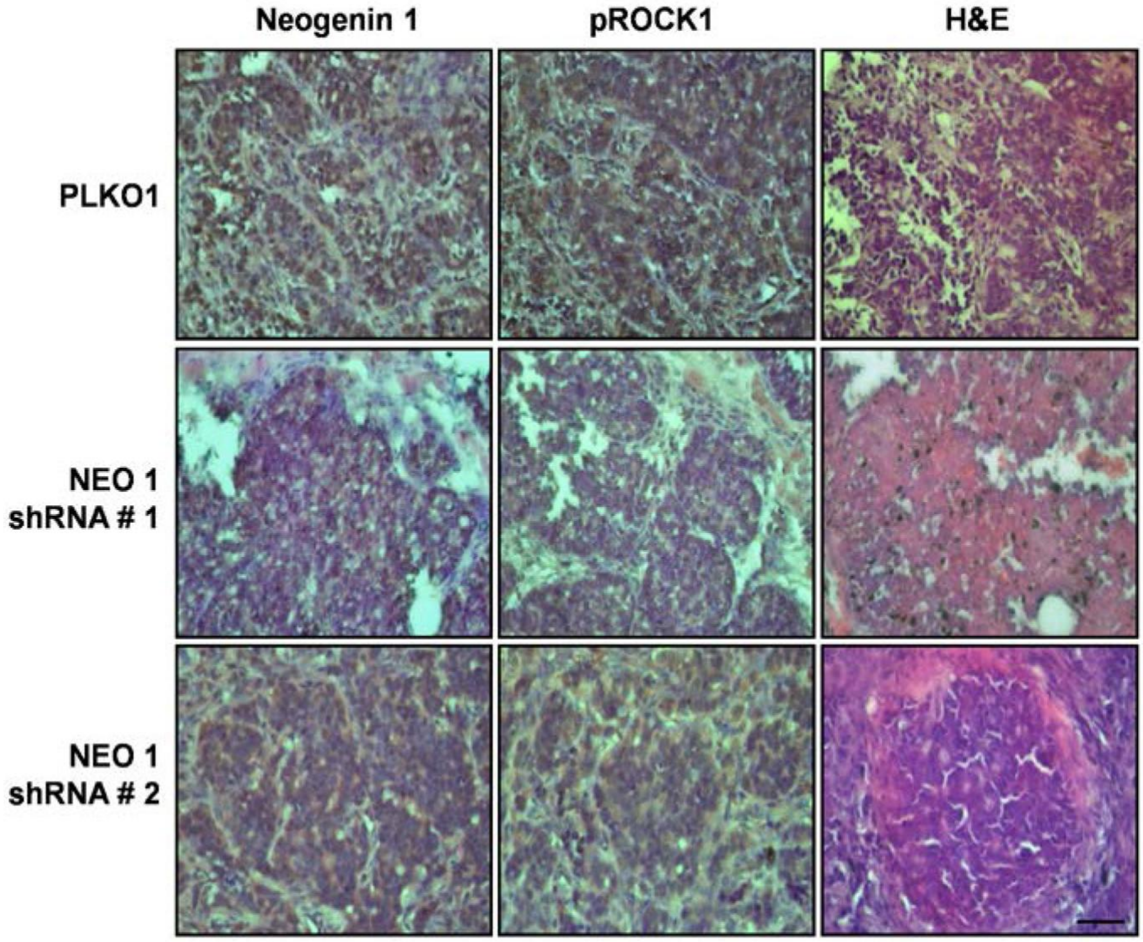

Supplementary Figure 4: Immunohistochemical analysis of ROCK1 phosphorylation by neogenin-1 depleted tumors of AGS xenografted mice. Immunohistochemical analysis and H&E staining were performed for each group of tumors, as described in “Methods” (magnification X100).

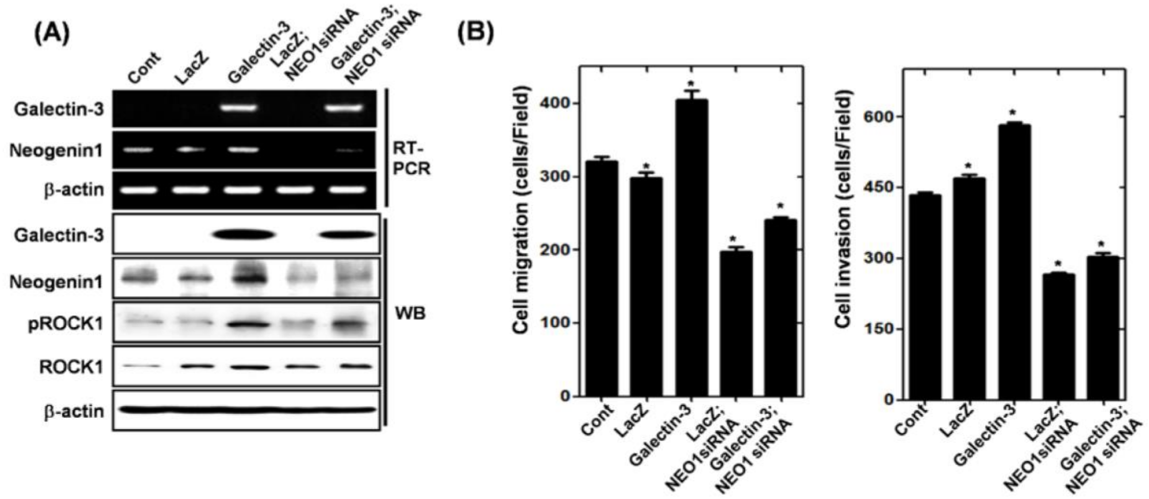

Supplementary Figure 5: Galectin-3-mediated neogenin-1 expression promotes gastric cancer cell invasion through ROCK1 activation. (A) Expression levels of galectin-3 and neogenin-1 were detected by RT-PCR and Western blotting analysis in SNU-638 cells, which were infected with lentivirus containing LacZ or galectin-3, and by subsequent transfection with neogenin-1 siRNA or scRNA. β-actin was used as a loading control. **(B)** These cells were analyzed by migration assay and invasion assay, and results are presented as histograms (* $p<0.0001$ vs. Cont; one-way ANOVA).

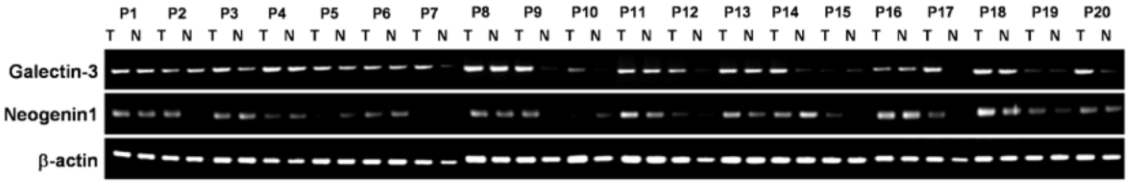

Supplementary Figure 6: mRNA expression levels of galectin-3 and neogenin-1 were analyzed by RT-PCR in 20 gastric cancer patients. Malignant and normal tissue pairs were obtained from each of 20 gastric patients and RT-PCR analysis was performed as described in the "Methods." β-actin was used as a normalization control.
